# Targeting sphingosine kinase 2 (SphK2) by ABC294640 inhibits colorectal cancer cell growth *in vitro* and *in vivo*

**DOI:** 10.1186/s13046-015-0205-y

**Published:** 2015-09-04

**Authors:** Cai Xun, Min-Bin Chen, Li Qi, Zhang Tie-Ning, Xue Peng, Li Ning, Chen Zhi-Xiao, Wang Li-Wei

**Affiliations:** Department of Oncology, Shanghai General Hospital, Shanghai Jiaotong University, 100 Haining Road, Shanghai, Hongkou District 200080 China; Department of Oncology, Shanghai First People’s Hospital, Nanjing Medical University, Shanghai, China; Department of Oncology, Kunshan First People’s Hospital Affiliated to Jiangsu University, Kunshan, China

**Keywords:** Colorectal cancer, Sphingosine kinase, ABC294640, Ceramide and signaling

## Abstract

**Background:**

Colorectal cancer (CRC) is a major health problem in China and around the world. It is one of the leading causes of cancer-related deaths. Research groups are thus searching for novel and more efficient anti-CRC agents.

**Results:**

Here we demonstrated that ABC294640, a novel SphK2 inhibitor, induced growth inhibition and apoptosis in transformed and primary CRC cells. The SphK activity was remarkably inhibited by ABC294640, accompanied by sphingosine-1-phosphate (S1P) depletion and ceramide incensement in CRC cells. Exogenously-added S1P inhibited ABC294640-induced HT-29 cell lethality. While C6 ceramide and SphK1 inhibitor SKI-II facilitated ABC294640-induced cytotoxicity against HT-29 cells. ABC294640 inhibited AKT-S6K1, but activated JNK signaling in transformed and primary CRC cells. JNK inhibitors (SP600125 and JNKi-II) alleviated ABC294640-induced CRC cell apoptosis. Moreover, a low concentration of ABC294640 sensitized the activity of 5-FU and cisplatin *in vitro. In vivo*, ABC294640 oral administration dramatically inhibited HT-29 xenografts growth in nude mice.

**Conclusions:**

Targeting of SphK2 by ABC294640 potently inhibits CRC cell growth both *in vitro* and *in vivo*, ABC294640 could be developed as a novel therapeutic for the treatment of CRC.

**Electronic supplementary material:**

The online version of this article (doi:10.1186/s13046-015-0205-y) contains supplementary material, which is available to authorized users.

## Background

Colorectal cancer (CRC) is a major health problem in China and around the world [1, 2]. It is one of the leading causes of cancer-related deaths [1, 2]. Over the past decades, significant improvements have been accomplished in chemotherapy treatments for CRC [1–3]. However, for those with advanced/malignant CRC, the overall survival has not been remarkably prolonged [4]. Research groups are thus searching for novel and more efficient anti-CRC agents [1, 2, 5–7].

Existing evidences have confirmed sphingosine kinase (SphK) as an important therapeutic target for CRC and other solid tumors [8]. SphK controls the balance of cellular sphingolipids [9–11]. Activation of SphK leads to generation of sphingosine-1-phosphate (S1P), which is a known lipid signaling molecule promoting several pro-cancer behaviors, including migration, differentiation, survival, angiogenesis and immune cell modulation [12]. On the other hand, SphK inactivation will induce accumulation of S1P precursors, including sphingosine and ceramide, causing cell apoptosis and growth arrest [13].

Thus far, there are at least two isoforms of SphK, SphK1 and SphK2, have been identified [14]. The oncogenic role of SphK1 has been extensively studied in CRC and other cancers [15]. Studies have demonstrated the pivotal role of SphK1 in cellular proliferation, survival, and its ability to reverse chemoresistance in CRC [15]. However, few is known about the role of SphK2 in CRC. It has been shown that the ablation of SphK2 by RNA interference (RNAi) inhibited cell proliferation and migration more effectively than that of SphK1 [16]. In CRC cells, SphK2 siRNA downregulation facilitated sodium butyrate-induced apoptosis [17].

Recent studies have characterized a novel SphK2 inhibitor, ABC294640, which was shown to suppress growth of breast, kidney, and pancreatic cancer cells [18–20]. ABC294640 is a non-lipid competitive inhibitor of SphK2, and exhibited chemotherapeutic pharmacological efficacy in animal models without systemic toxicity [18–20]. However, the potential effect of ABC294640 in CRC, and the underlying signaling mechanisms have been largely unknown. In this study, we used ABC294640 as a pharmacological tool to determine the efficacy of targeting SphK2 as a novel therapeutic intervention in the treatment of CRC.

## Methods

### Chemicals and antibodies

ABC294640 was purchased from DC Chemicals (Shanghai, China). SKI-II (4-[[4-(4-Chlorophenyl)-2-thiazolyl]amino]phenol) was purchased from Tocris Bioscience (Ellisville, Mo). Cell permeable short-chain C6 ceramide was obtained from Avanti Polar Lipids, Inc. (Alabaster, AL). S1P was purchased from Cayman Chemical Co. (Ann Arbor, MI). 5-fluorouracil (5-FU) and cisplatin were purchased from Sigma (Shanghai, China). SP600125 and JNK inhibitor II (JNKi-II) were obtained from Selleck (Shanghai, China). Antibodies against phospho (p)-AKT (Ser 473), p-AKT (Thr 308), p-ribosomal protein S6 kinase 1 (S6K1) (Thr-389), p-JNK1/2 (Thr 183/Tyr 185) and p-c-Jun (Ser73) were purchased form Cell Signaling Tech (Denver MA). Anti-AKT1, SphK2, c-Jun and tubulin antibodies were obtained from Santa Cruz (Santa Cruz, CA).

### CRC cell lines and culture

CRC cell lines, including HT-29, HCT-116 and DLD-1, were from the Cell Bank of CAS (Shanghai, China), cells were cultured in RPMI/DMEM medium, with a 10 % FBS in a CO_2_ incubator at 37 °C.

### Primary colon cancer cell isolation and culture

Surgery-isolated colon cancer tissues were thoroughly washed, non-cancerous surrounding tissues, if any, were separated carefully under microscopy, and were discarded. Clinical pathology reports confirmed those tissues were indeed colon cancer tissues. Tissues were then minced. The pellets were thoroughly washed, then re-pelleted at 400 g for 5 min, and were subjected to 0.15 % (w/v) collagenase I digestion for 1 hour. Primary cells were pelleted and rinsed twice with DMEM, cells were then cultured in medium (DMEM, 15 % FBS, 10 mg/ml transferrin, 2 mM glutamine, 1 mM pyruvate, 10 mM HEPES, 100 units/ml penicillin/streptomycin, 0.1 mg/ml gentamicin, 0.2 units/ml insulin, 0.1 mg/ml hydrocortisone, and 2 g/liter fungizone) [21]. Written informed consents were obtained from all enrolled patients. All clinical investigations were in accordance with principles expressed in the Declaration of Helsinki.

### MTT assay of cell proliferation

CRC cell proliferation was analyzed by the 3-[4, 5-dimethylthiazol-2-yl]-2, 5 diphenyltetrazolium bromide (MTT) assay. Briefly, 3,000 cells/well were plated in 96-well plates. After treatment, 20 μl/well of MTT (5 mg/ml, Sigma) was added to culture medium for 2 hours. Absorbance was measured on a microplate reader (Bio-Rad, Basel, Switzerland) at 570 nm.

### Clonogenic survival assay

SKOV3 cells were plated in 6-well plates at 1000 cells per well. Cells were then treated with gradient concentrations of ABC294640. Ten days after treatment, survival colonies were fixed with 3 % glutaraldehyde, 0.2 % crystal violet and 20 % methanol, and were manually counted.

### Annexin V FACS assay of cell apoptosis

Apoptosis was detected by an Annexin-V-FITC apoptosis detection kit (BD Pharmingen, San Diego, CA). Briefly, CRC cells were harvested and washed twice (with PBS), and then incubated for 15 min with Annexin-V-FITC and propidium iodide (PI). Both early (Annexin V^+^/PI^−^) and late (Annexin V^+^/PI^+^) apoptotic cells were sorted by the fluorescence activated cell sorter (FACS) machine (Becton Dickinson FACS Calibur, San Jose, CA). The percentage of Annexin V stained cells was gated as a quantitative measurement of cell apoptosis.

### Fragmented DNA detection by ELISA

Nucleosomal DNA fragmentation is one of the biological markers for apoptosis. Fragmented DNA was assessed by measuring DNA-associated with nucleosomal histones using a specific two-site ELISA with an anti-histone primary antibody and a secondary anti-DNA antibody, according to the manufacturer's instructions (Roche Applied Science, Shanghai, China). ELISA OD at 450 nm was recorded to measure cell apoptosis.

### Lactate dehydrogenase (LDH) assay

LDH content released into conditional medium indicates the level of toxicity. LDH content was assayed by a LDH detection kit from Roche Applied Science (Shanghai, China). % LDH release = LDH released in conditional medium/(LDH released in conditional medium + LDH in cell lysates)* 100 %.

### Western blots

Thirty μg of proteins per sample were separated by 10 % SDS-PAGE gel, and were transferred to polyvinylidene difluoride (PVDF) membranes (Millipore, Bedford, MA). After blocking with 10 % non-fat dry milk (in PBST) for 1 hour, membranes were incubated with designed antibodies (in PBST) overnight at 4 °C, followed by incubation with secondary antibodies (in PBST) for 1–2 hours at room temperature. The blots were visualized with enhanced chemiluminescence (ECL) kit (Pierce, Shanghai, China). The intensity of each band was quantified through ImageJ software after normalized to corresponding loading control.

### Assay of SphK activity and S1P content

After treatment, 20 μg of cell lysates were incubated with 25 μmol/L D-erythrosphingosine dissolved in 0.1 % Triton X-100, 2 mmol/L ATP, and [γ-32P] ATP for 30 minutes at 37 °C in a final volume of 200 μL. The reaction was stopped by adding 20 μL of HCl (1 N), followed by 800 μL of chloroform/methanol/HCl (100:200:1, v/v). After vigorous vortex, phases were separated by centrifugation. Radio-labeled S1P was separated by 60 thin-layer chromatography (TLC) on silica gel G60-plates with chloroform/acetone/methanol/acetic acid/water (10:4:3:2:1, v/v) as solvent, and phosphate incorporation was visualized and quantified using a scintillation counter (LS-6500, Beckman, Shanghai, China) [22]. The sphingosine kinase activity was valued as pmol/hour/g protein, and was expressed as percentage of the control group.

### Enzymatic measurement of ceramide

Cellular ceramide content in CRC cells was analyzed by the protocol reported in [23], and was valued as fmol by nmol of phospholipid. Its level in the treatment group was expressed as the percentage change of the control cells.

### HT-29 tumor bearing nude mice

A nude mice HT-29 xenograft experiment was performed to evaluate the *in vivo* activity of ABC294640. Animal care and procedures were in accordance with guidelines and regulations of the Institutional Animal Care and Use Committee (IACUC). This study is approved by the ethics committee of authors’ institutions. SKOV3 cells (2 × 10^6^ per mice) were implanted subcutaneously in right flanks, and tumor volumes were calculated by use of the equation: (L × W^2^)/2. When the tumors reached around 100 mm^3^, mice were randomly assigned to three groups. Treatment was then administered every day thereafter, consisting of oral doses of 5 or 20 mg of ABC294640/kg body weight or vehicle (0.375 % Polysorbate-80). Mice body weight and tumor volume measurements were performed every week. On week 6, tumors were excised, and weighted.

### Statistics analysis

Experiments in this study were repeated at least three times. Data were expressed as mean values ± standard deviations (SD). Statistics were analyzed by ANOVA followed by the Tukey’s multiple comparison (SPSS 18.0, Chicago, CA); The level of significance was P < 0.05. Cell doubling time was also calculated by SPSS software.

## Results

### ABC294640 induces growth inhibition and apoptosis in human CRC cell lines

**Fig. 1:**
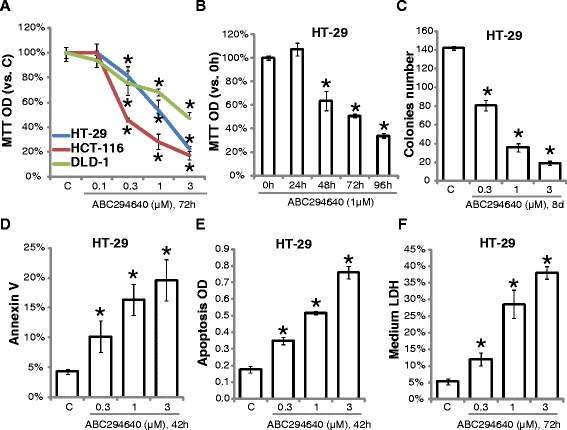
ABC294640 induces growth inhibition and apoptosis in CRC cell lines. The relative growth (vs. Control group) of tested CRC cells with indicated ABC294640 treatment was tested by MTT assay (**a** and **b**). HT-29 cells, treated with or without applied ABC294640 for indicated time, were subjected to clonogenicity assay (**c**), Annexin V FACS assay (**d**), histone-DNA ELISA assay (**e**) or LDH release assay (**f**). Mean values ± SD of three independent experiments were reported. Statistical analysis was performed comparing treatment groups with vehicle control group (“C”). *P < 0.05

We first examined the potential effect of ABC294640 on CRC cell growth. CRC cell lines, including HT-29, HCT-116 and DLD-1, were treated with applied concentrations of ABC294640, cell growth was tested by MTT assay. Results showed that ABC294640 dose-dependently inhibited CRC cell growth (Fig. 1a). The effect of ABC294640 was also time-dependent, and it took at least 48 hours for ABC294640 to exert the anti-proliferative activity in HT-29 cells (Fig. 1b). Meanwhile, ABC294640 treatment decreased the number of survival HT-29 colonies, further confirming its growth-inhibitory and cytotoxic activities (Fig. 1c). Next, we tested the role of ABC294640 on HT-29 cell apoptosis. Results of Annexin V FACS assay (Fig. 1d) and histone-DNA ELISA assay (Fig. 1e) demonstrated that ABC294640 induced significant apoptosis in HT-29 cells. Meanwhile, LDH content in conditional medium of ABC294640-treated cells was also increased (Fig. 1f). Similar apoptosis and LDH results were also seen in two other CRC cell lines (HCT-116 and DLD-1) (Data not shown). Together, these results show that ABC294640 induces growth inhibition and apoptosis in cultured CRC cells.

### ABC294640 decreases SphK activity, causing S1P depletion and ceramide accumulation in CRC cells

**Fig. 2:**
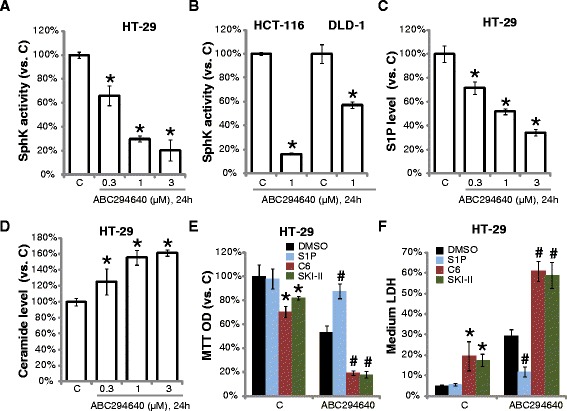
The effect of ABC294640 on SphK activity, S1P or ceramide content in CRC cells. The relative SphK activity (**a** and **b**), S1P content (**c**) or ceramide level (**d**) (vs. Control group) with indicated ABC294640 treatment were presented. The effect of S1P (5 μM), C6 ceramide (C6, 5 μM) or SKI-II (5 μM) on ABC294640 (1 μM)-induced HT-29 cell growth inhibition and cell death were tested by MTT assay (**e**) and LDH release assay (**f**), respectively. Mean values ± SD of three independent experiments were reported. Statistical analysis was performed comparing treatment groups with vehicle control group (“C”). *P < 0.05. ^#^P < 0.05 vs. ABC294640 only group (**e** and **f**)

Next, we tested the SphK activity in ABC294640-treated CRC cells. As shown in Fig. 2a, ABC294640 at tested concentrations remarkably inhibited SphK activity in HT-29 cells. Further, SphK activity was also decreased in ABC294640-treated HCT-116 cells and DLD-1 cells (Fig. 2b). As a consequence, the S1P content was decreased by ABC294640 in HT-29 cells (Fig. 2c), and the cellular ceramide level was increased (Fig. 2d). Note that, the expression level of SphK2 (tested by Western blots) was not affected by above ABC294640 treatment (Data not shown). Significantly, exogenously-added S1P alleviated ABC294640-induced growth inhibition and apoptosis in HT-29 cells (Fig. 2e and f). Conversely, a short-chain ceramide (C6) and the SphK1 inhibitor SKI-II exacerbated ABC294640-induced HT-29 cytotoxicity (Fig. 2e and f). Ceramide (C6) or the SKI-II alone also induced obvious cytotoxicity in HT-29 cells (Fig. 2e and f). These results indicate that ABC294640-induced anti-CRC activity *in vitro* is accompanied with SphK inactivation, S1P depletion and ceramide accumulation.

### ABC294640 was cytotoxic to primary human CRC cells

**Fig. 3:**
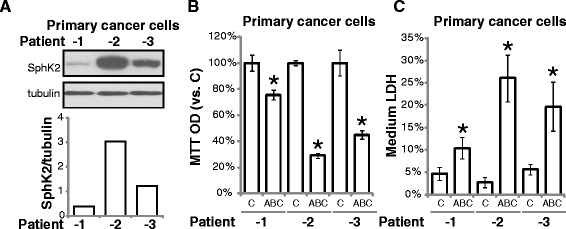
The cytotoxic effect of ABC294640 in primary human CRC cells. *Ex vivo* cultured primary cancer cells derived from three different colon cancer patients (−1, male, 47 yeas old; −2, female, 62 years old; −3, male, 55 years old) were treated with or without ABC294640 (1 μM) for 72 hours, cell growth and cell death were tested by MTT assay (**b**) and LDH release assay (**c**), respectively. Expression of SphK2 and tubulin in above cells was also shown (**a**). Mean values ± SD of three independent experiments were reported. Statistical analysis was performed comparing treatment groups with vehicle control group (“C”). *P < 0.05

The activity of ABC294640 on patient-derived primary cancer cells was tested. We successfully cultured primary cancer cells from 3 different colon cancer patients. Their corresponding *in vitro* growth curve and doubling time were presented in Additional file 1: Figure S1A and B. As shown in Fig. 3a, these primary cancer cells showed differential expression of SphK2 expression. Patient-2-derived cancer cells had highest level of SphK2, these cells were extremely sensitive to ABC294640-induced growth inhibition (Fig. 3b) and cell death (Fig. 3c). On the other hand, patient-1-derived cancer cells had lowest SphK2 level (Fig. 3a), the cytotoxic effect of ABC29464 was also relatively weak in those cells (Fig. 3b and c). Thus, ABC294640 is cytotoxic to the tested primary cancer cells, and its activity is negatively associated with SphK2 expression level. The morphology of these primary cancer cells before and after ABC294640 treatment was shown in Additional file 1: Figure S1C.

### ABC294640 inactivates AKT-S6K1, but activates JNK signaling in cultured CRC cells

**Fig. 4:**
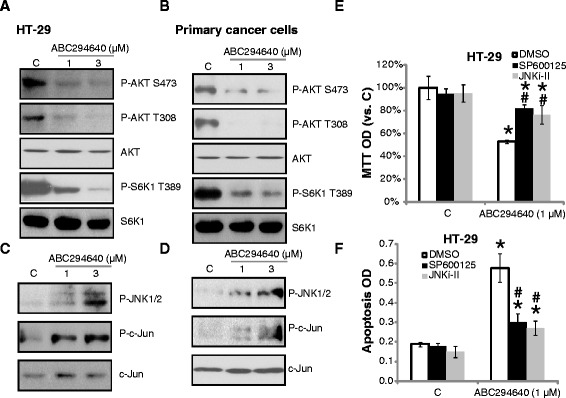
Signaling changes in ABC294640-treated CRC cells. HT-29 cells or primary CRC cells were treated with or without ABC294640 (1/3 μM) for 12 hours, listed proteins were tested by Western blots (**a**-**d**). HT-29 cells were pre-treated with SP600125 (5 μM) or JNK inhibitor II (JNKi-II, 5 μM) for 1 hour, followed by ABC294640 (1 μM) stimulation, cell growth (**e**) and cell apoptosis (**f**) were analyzed. Mean values ± SD of three independent experiments were reported. Statistical analysis was performed comparing treatment groups with vehicle control group (“C”). *P < 0.05. ^#^P < 0.05 vs. ABC294640 only group (**e** and **f**)

Activation of AKT-mammalian target of rapamycin (mTOR) signaling has been linked to CRC cell survival, proliferation and chemo-resistance [24]. Next, we tested the effect of ABC294640 on AKT-mTOR activation in CRC cells. Activation of AKT was evidence by phosphorylated (p-) AKT at both Ser-473 and Thr-308. Western blot results in Fig. 4a showed that ABC294640 at 1 μM or 3 μM remarkably inhibited AKT phosphorylation at both sites in HT-29 cells. S6K1 phosphorylation, an indicator of mTOR complex C1 (mTORC1) activation, was also dramatically inhibited by ABC294640 (Fig. 4a). Expression of regular AKT and S6K1 was not affected by the same ABC294640 treatment (Fig. 4a). Similar results were also achieved in primary CRC cells (Fig. 4b). Meanwhile, activation of JNK, tested by p-JNK1/2 and p-c-Jun, was induced by same ABC294640 treatment in HT-29 cells (Fig. 4c), and in primary CRC cells (Fig. 4d). Notably, the JNK inhibitors, SP600125 and JNKi-II, suppressed ABC294640-induced HT-29 growth inhibition (Fig. 4e) and apoptosis (Fig. 4f), indicating a pro-apoptotic role of JNK activation by ABC294640 in CRC cells. Similar results were also seen in primary cancer cells (Data not shown).

### ABC294640 sensitizes the activity of 5-FU and cisplatin

**Fig. 5:**
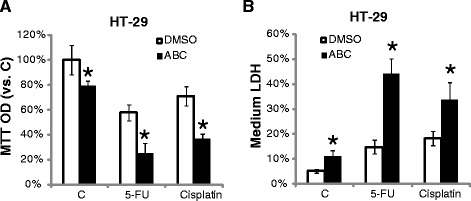
The *in vitro* chemo-sensitization activity by ABC294640. HT-29 cells were treated with 5-FU (5 μM), cisplatin (5 μM), or together with ABC294640 (0.3 μM) for 72 hours, cell growth was tested by MTT assay (**a**), and cell death was evaluated by LDH release assay (**b**). Mean values ± SD of three independent experiments were reported. Statistical analysis was performed comparing groups with or without ABC294640 co-treatment. *P < 0.05

Chemoresistance is major problem in current CRC treatment [1, 2]. The results above showed that ABC294640 treatment induced SphK2 and AKT-mTOR inactivation, S1P depletion, and ceramide accumulation in CRC cells. All these should favor a chemo-sensitization consequence [1, 2]. Thus, we tested the potential effect of ABC294640 on 5-fluorouracil (5-FU) and cisplatin, two widely utilized anti-CRC chemo-drugs, in CRC cells [1, 2]. As shown in Fig. 5a, 5-FU or cisplatin alone at tested concentration only induced moderate growth inhibition in HT-29 cells, co-administration with a low concentration of ABC294640 (0.3 μM) dramatically enhanced their sensitivities (Fig. 5a). Meanwhile, LDH results showed that ABC294640 remarkably facilitated 5-FU and cisplatin-induced HT-29 cell death (Fig. 5b). Note that similar chemo-sensitization effect by ABC294640 was also reproduced in two other CRC cell lines (HCT-116 and DLD-1) (Data not shown). These results demonstrate the 5-FU/cisplatin sensitization effect by ABC294640 in cultured CRC cells.

### Oral administration of ABC294640 inhibits HT-29 xenografts growth in nude mice

**Fig. 6:**
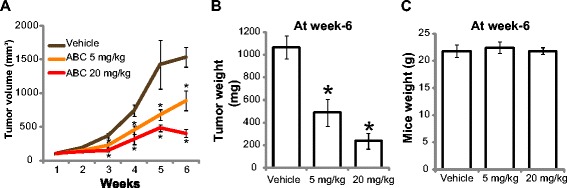
ABC294640 inhibits HT-29 cell growth *in vivo*. The volumes of HT-29 xenograft tumors in nude mice administrated with ABC294640 (ABC, 5 or 20 mg/kg, *p.o.*, daily) or vehicle (0.375 % Polysorbate-80) control were recorded weekly (n = 10 for each group) (**a**). Tumor weights (**b**) and mice body weights (**c**) at week-6 were also presented. Statistical analysis was performed comparing treatment groups with vehicle control group. *P < 0.05

At last, we studied the anti-CRC activity of ABC294640 *in vivo.* HT-29 tumor bearing nude mice model was applied. Established, sized-matched HT-29 tumors were divided into three groups: low-dose ABC294640 (5 mg/kg, daily, *p.o.*) treatment group (n = 10), high-dose ABC294640 (20 mg/kg, daily, *p.o.*) treatment group (n = 10), and the vehicle (0.375 % Polysorbate-80) control group (n = 10). Tumor growth curve results in Fig. 6a showed that oral administration of ABC294640 (5 or 20 mg/kg) dramatically inhibited HT-29 xenograft growth in nude mice. The weights ABC294640-treated tumors were remarkably lower than that of vehicle-treated group (Fig. 6b). Mice body weights were not affected by ABC294640 administration throughout the experiment duration (see the data of week-6 in Fig. 6c). Nor did we noticed any signs of system toxicity or wasting. Thus, in line with the *in vitro* results, oral administration of ABC294640 dramatically inhibits HT-29 cell growth *in vivo.*

## Discussions

SphK has been recognized as an important therapeutic target in various solid tumors, mainly due to its roles in cell proliferation, survival, apoptosis, differentiation, and cell senescence [8, 14, 25, 26]. Thus far, at least two isoforms of SphK have identified, including SphK1 and SphK2 [14]. The two have various tissue expression, and subcellular localization [25, 27]. It has been shown that SphK1 expression is highest in lung, spleen, kidney, and blood; Meanwhile, overexpression of SphK2 is found in liver, kidney, brain, and heart [28, 29]. In the current study, we showed that SphK2 expression level was inconsistent in different primary colon cancer cells, which negatively correlated to ABC294640’s sensitivity. Although the role of SphK1 in cancer progression has been extensively studied in the literature [26], the function of SphK2 on cell survival, growth and drug resistance has only recently been elucidated [30, 31].

In the current study, our results showed that ABC294640, the novel, specific and competitive SphK2 inhibitor, suppressed SphK activation in CRC cells, causing S1P depletion and ceramide accumulation. ABC294640 inhibited CRC cell growth, and induced CRC cell apoptosis. Further, it sensitized 5-FU- and cisplatin-mediated anti-HT-29 cell activity. *In vivo*, oral administration of ABC294640 remarkably inhibited HT-29 xenografts growth in nude mice, without inducing system toxicity. These preclinical results suggest that ABC294640 might be an efficient anti-CRC agent.

One of the novel findings of this study is that ABC294640 significantly inhibited AKT-mTOR (S6K1) activation in both transformed and primary CRC cells. Existing evidence have confirmed the pivotal role of AKT-mTOR signaling in regulating CRC cancer cell growth, proliferation, migration and survival. This pathway is frequently dysregulated in CRC [24, 32, 33]. Thus, AKT-mTOR inhibition by ABC294640 might be responsible, at least in part, for its cytotoxic effects against CRC cells. These results are not surprising, since SphK2 blockage by ABC294640 caused ceramide accumulation in CRC cells. Ceramide is able to activate phosphatase 1A (PP1A) or PP2 to directly de-phosphorylate AKT [34, 35]. S1P was shown to activate AKT through different mechanisms [36], and decreased S1P in ABC294640-treated cells might also be the reason of AKT-mTOR inactivation. The detailed signaling mechanisms of AKT-mTOR inhibition by this SphK2 inhibitor require further characterizations.

Another important signaling discovery of this study is JNK activation by ABC294640 in tested CRC cells, which is also involved in ABC294640-mediated activity. Inhibition of JNK by two different JNK inhibitors alleviated growth inhibition and apoptosis by ABC294640. Although the detailed signaling mechanisms need further investigation, there are possible speculations to explain JNK activation by ABC294640. Chen et al., showed that ceramide alone induced JNK activation and subsequent cell apoptosis through thioredoxin-interacting protein-mediated pathway [37]. SiRNA-mediated silencing of JNK largely reversed ceramide-induced apoptosis [37]. Based on these information, it is possible that blockage of SphK2 by ABC294640 leads to ceramide accumulation, which actives JNK signaling to promote CRC cell apoptosis.

One advantage of using of ABC294640, other than it is a novel, highly-efficient and competitive SphK2 inhibitor, is its oral availability. In the current study, our results demonstrated that oral administration of a single dose of ABC294640 (5 or 20 mg/kg, daily) dramatically inhibited HT-29 xenograft growth in nude mice, leading to remarkable tumor recession. That’s being said, however, certain off-target effects of ABC294640 should be considered. For example, it has been previously shown that ABC294640 binds to the E2 estrogen receptor (ER) in an antagonistic manner, and similar to tamoxifen, this novel SphK2 inhibitor represses ER signaling in both cellular and animal models [31]. Although this is somehow advantageous to ER-expressing breast cancer cells [31], yet ER inhibition and other potential side effects should be considered fully when given to human, and close monitoring of patients is thus recommended.

## Conclusions

Thus, targeting of SphK2 by ABC294640 potently inhibits CRC cell growth both *in vitro* and *in vivo*, ABC294640 could be developed as a novel therapeutic for the treatment of CRC.

## Additional file

Additional file 1: Figure S1. Same amount (1*10^3^/dish) of the primary colon cancer cells were cultured in FBS-containing medium for applied time, number of viable cells (trypan blue exclusive) at each time point was presented (**A**), their doubling-time was calculated, and was compared to that of HT-29 cells (**B**). Above primary cancer cells were treated with or without ABC294640 (1 μM) for 72 hours, representative morphology images were shown (**C**). Mean values ± SD of three independent experiments were reported. Magnification: 1: 200 (**C**). (EPS 4507 kb)

## References

[1] Schmoll HJ, Stein A (2014). Colorectal cancer in 2013: Towards improved drugs, combinations and patient selection. Nat Rev Clin Oncol.

[2] Palta M, Czito BG, Willett CG (2014). Colorectal cancer: adjuvant chemotherapy for rectal cancer-an unresolved issue. Nat Rev Clin Oncol.

[3] Tonini G, Imperatori M, Vincenzi B, Frezza AM, Santini D (2013). Rechallenge therapy and treatment holiday: different strategies in management of metastatic colorectal cancer. J Exp Clin Cancer Res.

[4] Siegel R, Ma J, Zou Z, Jemal A (2014). Cancer statistics, 2014. CA Cancer J Clin.

[5] Yang K, Jiang L, Hu Y, Yu J, Chen H, Yao Y, Zhu X (2015). Short hairpin RNA- mediated gene knockdown of FOXM1 inhibits the proliferation and metastasis of human colon cancer cells through reversal of epithelial-to-mesenchymal transformation. J Exp Clin Cancer Res.

[6] Xu JM, Liu XJ, Ge FJ, Lin L, Wang Y, Sharma MR, Liu ZY, Tommasi S, Paradiso A (2014). KRAS mutations in tumor tissue and plasma by different assays predict survival of patients with metastatic colorectal cancer. J Exp Clin Cancer Res.

[7] Nuvoli B, Santoro R, Catalani S, Battistelli S, Benedetti S, Canestrari F, Galati R (2014). CELLFOOD induces apoptosis in human mesothelioma and colorectal cancer cells by modulating p53, c-myc and pAkt signaling pathways. J Exp Clin Cancer Res.

[8] Pyne S, Bittman R, Pyne NJ (2011). Sphingosine kinase inhibitors and cancer: seeking the golden sword of Hercules. Cancer Res.

[9] Young MM, Kester M, Wang HG (2013). Sphingolipids: regulators of crosstalk between apoptosis and autophagy. J Lipid Res.

[10] Ogretmen B, Hannun YA (2004). Biologically active sphingolipids in cancer pathogenesis and treatment. Nat Rev Cancer.

[11] Gangoiti P, Granado MH, Alonso A, Goni FM, Gomez-Munoz A (2008). Implication of ceramide, ceramide 1-phosphate and sphingosine 1-phosphate in tumorigenesis. Transl Oncogenomics.

[12] Maceyka M, Harikumar KB, Milstien S, Spiegel S (2012). Sphingosine-1-phosphate signaling and its role in disease. Trends Cell Biol.

[13] Dimanche-Boitrel MT, Rebillard A, Gulbins E (2011). Ceramide in chemotherapy of tumors. Recent Pat Anticancer Drug Discov.

[14] Maceyka M, Payne SG, Milstien S, Spiegel S (2002). Sphingosine kinase, sphingosine-1-phosphate, and apoptosis. Biochim Biophys Acta.

[15] Shida D, Takabe K, Kapitonov D, Milstien S, Spiegel S (2008). Targeting SphK1 as a new strategy against cancer. Curr Drug Targets.

[16] Gao P, Smith CD (2011). Ablation of sphingosine kinase-2 inhibits tumor cell proliferation and migration. Mol Cancer Res.

[17] Xiao M, Liu Y, Zou F (2012). Sensitization of human colon cancer cells to sodium butyrate-induced apoptosis by modulation of sphingosine kinase 2 and protein kinase D. Exp Cell Res.

[18] French KJ, Zhuang Y, Maines LW, Gao P, Wang W, Beljanski V, Upson JJ, Green CL, Keller SN, Smith CD (2010). Pharmacology and antitumor activity of ABC294640, a selective inhibitor of sphingosine kinase-2. J Pharmacol Exp Ther.

[19] Gao P, Peterson YK, Smith RA, Smith CD (2012). Characterization of isoenzyme-selective inhibitors of human sphingosine kinases. PLoS One.

[20] Gestaut MM, Antoon JW, Burow ME, Beckman BS (2014). Inhibition of sphingosine kinase-2 ablates androgen resistant prostate cancer proliferation and survival. Pharmacol Rep.

[21] Li C, Cui JF, Chen MB, Liu CY, Liu F, Zhang QD, Zou J, Lu PH (2015). The preclinical evaluation of the dual mTORC1/2 inhibitor INK-128 as a potential anti-colorectal cancer agent. Cancer Biol Ther.

[22] Altura BM, Shah NC, Shah GJ, Zhang A, Li W, Zheng T, Perez-Albela JL, Altura BT (2014). Short-term Mg deficiency upregulates protein kinase C isoforms in cardiovascular tissues and cells; relation to NF-kB, cytokines, ceramide salvage sphingolipid pathway and PKC-zeta: hypothesis and review. Int J Clin Exp Med.

[23] Gong L, Yang B, Xu M, Cheng B, Tang X, Zheng P, Jing Y, Wu GJ (2014). Bortezomib-induced apoptosis in cultured pancreatic cancer cells is associated with ceramide production. Cancer Chemother Pharmacol.

[24] Zhang YJ, Dai Q, Sun DF, Xiong H, Tian XQ, Gao FH, Xu MH, Chen GQ, Han ZG, Fang JY (2009). mTOR signaling pathway is a target for the treatment of colorectal cancer. Ann Surg Oncol.

[25] Zhang Y, Wang Y, Wan Z, Liu S, Cao Y, Zeng Z (2014). Sphingosine kinase 1 and cancer: a systematic review and meta-analysis. PLoS One.

[26] Vadas M, Xia P, McCaughan G, Gamble J (1781). The role of sphingosine kinase 1 in cancer: oncogene or non-oncogene addiction?. Biochim Biophys Acta.

[27] Orr Gandy KA, Obeid LM (1831). Targeting the sphingosine kinase/sphingosine 1-phosphate pathway in disease: review of sphingosine kinase inhibitors. Biochim Biophys Acta.

[28] Kihara A, Anada Y, Igarashi Y (2006). Mouse sphingosine kinase isoforms SPHK1a and SPHK1b differ in enzymatic traits including stability, localization, modification, and oligomerization. J Biol Chem.

[29] Kohama T, Olivera A, Edsall L, Nagiec MM, Dickson R, Spiegel S (1998). Molecular cloning and functional characterization of murine sphingosine kinase. J Biol Chem.

[30] Yang J, Yang C, Zhang S, Mei Z, Shi M, Sun S, Shi L, Wang Z, Wang Y, Li Z, Xie C (2015). ABC294640, a sphingosine kinase 2 inhibitor, enhances the antitumor effects of TRAIL in non-small cell lung cancer. Cancer Biol Ther.

[31] Antoon JW, White MD, Meacham WD, Slaughter EM, Muir SE, Elliott S, Rhodes LV, Ashe HB, Wiese TE, Smith CD (2010). Antiestrogenic effects of the novel sphingosine kinase-2 inhibitor ABC294640. Endocrinology.

[32] Easton JB, Houghton PJ (2006). mTOR and cancer therapy. Oncogene.

[33] Ekstrand AI, Jonsson M, Lindblom A, Borg A, Nilbert M (2010). Frequent alterations of the PI3K/AKT/mTOR pathways in hereditary nonpolyposis colorectal cancer. Fam Cancer.

[34] Lin CF, Chen CL, Lin YS (2006). Ceramide in apoptotic signaling and anticancer therapy. Curr Med Chem.

[35] Sumanasekera C, Kelemen O, Beullens M, Aubol BE, Adams JA, Sunkara M, Morris A, Bollen M, Andreadis A, Stamm S (2012). C6 pyridinium ceramide influences alternative pre-mRNA splicing by inhibiting protein phosphatase-1. Nucleic Acids Res.

[36] Pyne NJ, Pyne S (2010). Sphingosine 1-phosphate and cancer. Nat Rev Cancer.

[37] Chen CL, Lin CF, Chang WT, Huang WC, Teng CF, Lin YS (2008). Ceramide induces p38 MAPK and JNK activation through a mechanism involving a thioredoxin-interacting protein-mediated pathway. Blood.

